# Comparative Effect of Lisinopril and Fosinopril in Mitigating Learning and Memory Deficit in Scopolamine-Induced Amnesic Rats

**DOI:** 10.1155/2015/521718

**Published:** 2015-08-02

**Authors:** Debasree Deb, K. L. Bairy, Veena Nayak, Mohandas Rao

**Affiliations:** ^1^Department of Pharmacology, Melaka Manipal Medical College, Manipal University, Manipal Campus, Manipal 576104, India; ^2^Department of Pharmacology, Kasturba Medical College, Manipal University, Manipal 576104, India; ^3^Department of Anatomy, Melaka Manipal Medical College, Manipal University, Manipal Campus, Manipal 576104, India

## Abstract

Lisinopril and fosinopril were compared on scopolamine-induced learning and memory deficits in rats. A total of eighty-four male Wistar rats were divided into seven groups. Group I received 2% gum acacia orally for 4 weeks, group II received normal saline, and group III received scopolamine (2 mg/kg/ip) as single dose. Groups IV and V received lisinopril ( 0.225 mg/kg and 0.45 mg/kg), while Groups VI and VII received fosinopril (0.90 mg/kg and 1.80 mg/kg), respectively, orally for four weeks, followed by scopolamine (2 mg/kg/ip) given 45 minutes prior to experimental procedure. Evaluation of learning and memory was assessed by using passive avoidance, Morris water maze, and elevated plus maze tests followed by analysis of hippocampal morphology and quantification of the number of surviving neurons. Scopolamine induced marked impairment of memory in behavioral tests which correlated with morphological changes in hippocampus. Pretreatment with fosinopril 1.80 mg/kg was found to significantly ameliorate the memory deficits and hippocampal degeneration induced by scopolamine. Fosinopril exhibits antiamnesic activity, indicating its possible role in preventing memory deficits seen in dementia though the precise mechanism underlying this effect needs to be further evaluated.

## 1. Introduction

Learning and memory are the most fundamental and closely related processes in the brain. Memory is defined as a change in mental representation caused by an experience, and learning is defined as a process of acquiring memory [1]. During this period of consolidation, memory can be disrupted with a wide variety of amnesia inducing agents. Scopolamine, a muscarinic receptor antagonist, induces memory deficits in rodents and healthy humans, and this effect has been proposed to mimic the cognitive and behavioral deficits seen during aging or in Alzheimer's disease (AD) [2]. Scopolamine produces a reversible impairment in maintaining attention, processing of information, and the acquisition of new knowledge in both rodents [3] and humans [4]. The amnesic action produced by the administration of scopolamine has thus been widely used as an experimental model for the screening and validation of drugs with potential cognitive enhancing ability [5, 6].

Angiotensin converting enzyme inhibitors (ACEIs) are a class of drugs effective in controlling hypertension and treating congestive heart failure, and their use in these patients has been associated with reduced cardiovascular morbidity and mortality [7]. However, in addition to their role in controlling blood pressure, ACE inhibitors have been shown to be effective in preventing cognitive decline and improving cognitive function in patients with hypertension [8, 9]. It has also been suggested that all ACE inhibitors do not prevent dementia in older adults being treated for hypertension but centrally acting ACEIs such as ramipril or perindopril do appear to reduce cognitive decline in older adults [10]. Because all ACEIs share a similar mechanism of action, it can be assumed that all centrally acting ACEIs may possess cognitive enhancing activity like ramipril or perindopril. The present study was thus undertaken to investigate the effects of two centrally acting ACEIs, namely, lisinopril and fosinopril, for their effect on learning and memory in scopolamine-induced amnesic rats. Further, the effects of scopolamine and test drugs on rat hippocampal morphology were analysed followed by quantification of the number of healthy neurons.

## 2. Materials and Methods

### 2.1. Animals

A total of eighty four male Wistar rats weighing 200–250 grams were used in the study. All animals were housed in polypropylene cage with only four animals in each cage to prevent overcrowding. The animals were kept at room temperature (25 ± 3°C) with a 12 h dark/light cycle and were provided with standard laboratory feed (VRK Nutritional Solutions, Pune, India Ltd.) and water* ad libitum*. The experimental protocol was approved by the Institutional Animal Ethical Committee (number IAEC/KMC/36/2011-2012, May 2011) and experiments were conducted in accordance with the CPSCEA guidelines on the use and care of experimental animals.

### 2.2. Drugs and Doses

Lisinopril and fosinopril powders were obtained as generous gift samples from Torrent Pharmaceuticals Ltd., Ahmedabad, India. Scopolamine hydrobromide was procured from Sigma Aldrich, Mumbai.

Rats equivalent doses in mg/kg body weight of clinical doses were calculated as mg/kg body weight as described by Paget and Barnes [11]. All the drugs except scopolamine were dissolved in 2% gum acacia while scopolamine was dissolved in normal saline.

The experiment was conducted in two stages as follows.

**Stage 1 .**

A total of 42 male Wistar rats were randomly divided into seven groups for assessing learning and memory using the elevated plus maze test and passive avoidance test.

**Stage 2 .**

A total of 42 male Wistar rats were randomly divided into seven groups for assessing learning and memory using the Morris water maze test.

The seven groups were divided as follows:

 Group I: 2 mL/kg of 2% gum acacia (normal control),
 Group II: 1 mL/kg of 0.9% normal saline i.p. (saline control),
 Group III: 2% gum acacia and scopolamine treatment,
 Group IV: lisinopril 0.225 mg/kg and scopolamine treatment,
 Group V: lisinopril 0.45 mg/kg and scopolamine treatment,
 Group VI: fosinopril 0.90 mg/kg and scopolamine treatment,
 Group VII: fosinopril 1.80 mg/kg and scopolamine treatment.

Each of the above groups of animals (except Group II) was treated orally for 4 weeks. Group II received i.p. injection of normal saline 45 minutes before experimental procedures. Scopolamine 2 mg/kg [12, 13] was administered intraperitoneally to the above groups of animals (except Groups I and II) for induction of amnesia, 45 minutes before the behavioural tests.

### 2.3. Behavioural Tests

#### 2.3.1. Elevated Plus Maze Test

Elevated plus maze serves as the exteroceptive behavioral model to evaluate acquisition and retention of memory in rats. The elevated plus maze for rats consists of two open arms (16 cm × 5 cm) and two covered arms (16 cm × 5 cm × 12 cm) extended from a central platform (5 cm × 5 cm) and is elevated to a height of 25 cm from the floor. Each rat was placed at the end of an open arm, facing away from the central platform. The rats received drug treatment for 4 weeks, followed by administration of scopolamine (2 mg/kg body weight, dissolved in normal saline) for induction of amnesia, 45 minutes before the training trial. Transfer latency (TL) which is the time taken by the rats to move from open arm to closed arm with all four legs in elevated plus maze was noted. The rat was allowed to explore the maze for another 2 min and then allowed to return to its home cage. After 24 hours of acquisition trial, TL was again noted as an index of retrieval [14].

#### 2.3.2. Step-Through Passive Avoidance Test

Passive avoidance test is an exteroceptive behavioural model for testing learning and memory in experimental rodents. The apparatus has a box (27 cm × 27 cm × 27 cm) of three wooden walls and one Plexiglas wall, with a grid floor (made up of 3 mm stainless-steel rods set 8 mm apart) and a platform (10 cm × 7 cm × 1.7 cm) at the centre of the grid floor. The box was kept illuminated with a 15 W bulb during the experiment. Each rat was kept in the larger compartment facing away from the entrance to the dark compartment. Three exploratory trials were given to each rat in which the rat explored the apparatus for 3 minutes. The intertrial interval was 5 minutes. The rat was removed from the cage during the intertrial period. In each trial, the total time taken by the animal to enter the dark compartment was noted using a stopwatch. A decrease in the latency to enter the dark compartment was considered as an index of improved learning. After the third exploratory trial, the rat was kept in the light compartment and when it entered the dark compartment, the sliding door was closed and three foot shocks (50 Hz, 1.5 mA, and 1 s duration) were delivered at 5-second intervals. The retention test was carried out after 24 hours of receiving the aversive stimuli.

Rats received gum acacia or test compounds for 4 weeks; this was followed by scopolamine (2 mg/kg body weight, dissolved in normal saline) for induction of amnesia, 45 minutes before the acquisition trial. After 24 hours of acquisition trials, the rats were again placed in the light compartment. The latency time required for the animal to enter the dark compartment and the total time spent by the animal in the light compartment were recorded. The latency time was recorded as 3 minutes for those animals that did not enter the dark compartment within 3 minutes. Increase in the latency to enter the dark compartment and more time spent in the light compartment indicated positive memory retention [15].

#### 2.3.3. Morris Water Maze Test

Morris water maze is a behavioural test to evaluate spatial learning and memory in experimental rodents. It is a circular tank (diameter 150 cm and height 45 cm), which was filled with water and maintained at 25°C. The water was made opaque by adding milk. The tank was divided into four equal quadrants (Q1, Q2, Q3, and Q4). A white platform (10 cm^2^) centered in one of the four quadrants of the pool was submerged approximately 1 cm below the surface of water. The position of platform and clues were kept consistent throughout the training session. In our study, the target quadrant was considered as Q4. Each animal was subjected to four consecutive acquisition trials on each day with an interval of 5 min, during which rats were allowed to locate the hidden platform and allowed to remain there for 20 sec. If the animal was unable to locate the hidden platform within 60 sec, it was gently guided by hand to the platform and allowed to remain there for 20 sec. During each trial, the latencies of rats to locate the hidden platform were recorded and the latency was considered as an index of acquisition and learning. On the 5th day, the platform was removed and each rat was allowed to explore the pool for 60 s. The latency to enter the target quadrant Q4 and the total time spent in target quadrant Q4 were noted as indices of retrieval. Rats received gum acacia or test compounds for 4 weeks; this was followed by scopolamine (2 mg/kg body weight, dissolved in normal saline) for induction of amnesia. All the animals were tested for spatial memory 45 mins after the scopolamine treatment [16, 17].

### 2.4. Histological Analysis by Hematoxylin and Eosin (H&E) Staining

All histological procedures were kept uniform for control and test group animals. At the end of behavioural tests, the rats were sacrificed by cervical dislocation under ether anaesthesia. The animals were perfused with 250 mL 4% paraformaldehyde, followed by 0.01 mol/L phosphate buffered saline (PBS), and the brain was exposed by cutting the skull along the midline. The brain section with hippocampus was carefully dissected out and fixed in 10% buffered formalin (with pH 7.4) for 24 h. The brains were then dehydrated in ascending grades of alcohol: 50% alcohol: 24 hours, 70% alcohol: 24 hours, 90% alcohol: 12 hours, and absolute alcohol: 12 hours. The tissue was cleared in xylene for 1-2 hours, infiltrated with paraffin wax (4 changes of 1 hour each), and embedded in fresh paraffin wax. Five-micron thick paraffin sections were obtained and mounted on clean glass slides, labeled, and stained with hematoxylin and eosin (H&E) according to standard procedure. The hippocampal CA1, CA3, and dentate gyrus regions were studied under a light microscope. To avoid observer's bias, an independent person coded the slides before subjecting them to morphological evaluations.

Quantification of neurons in the subregions of hippocampus (CA1, CA3, and dentate gyrus) was done using the light microscope under 40x (Magnus, Olympus Pvt. Ltd., New Delhi, India). To avoid manual bias slides from different groups were coded while counting. Cell counting was done using an ocular micrometer. The number of surviving or viable neurons (neurons with a distinct nucleus) within a specific measured (using ocular micrometer) area (e.g., 250-micron area) under 40x was counted. Ten sections were counted per rat and the mean was taken. Cells with darkly stained shrunken cell body and cells with fragmented nuclei were excluded from quantification.

### 2.5. Statistical Analysis

Data obtained from experiments were expressed as mean ± SE. Statistical differences between the treatment and the control groups were calculated by one-way analysis of variance (ANOVA) followed by Tukey's post hoc test. The data was considered to be statistically significant if the probability had a value of 0.05 or less.

## 3. Results

### 3.1. Effects of Lisinopril and Fosinopril on Elevated Plus Maze

Transfer latency (TL) of control animals decreased on the 2nd day, after 24 hours of training on the elevated plus maze. Administration of scopolamine increased the TL on the 1st and 2nd days, and it was significantly different compared to control (*p* < 0.001). Pretreatment with low and high doses of lisinopril and low dose of fosinopril did not show any significant difference in TL of rats on 1st and 2nd days compared to scopolamine group. Rats which were pretreated with high dose of fosinopril however showed a significant decrease in TL of rats on 1st and 2nd days compared to scopolamine group (*p* < 0.001), as shown in Table 1.

### 3.2. Effects of Lisinopril and Fosinopril in Passive Avoidance Test

**Figure 1:**
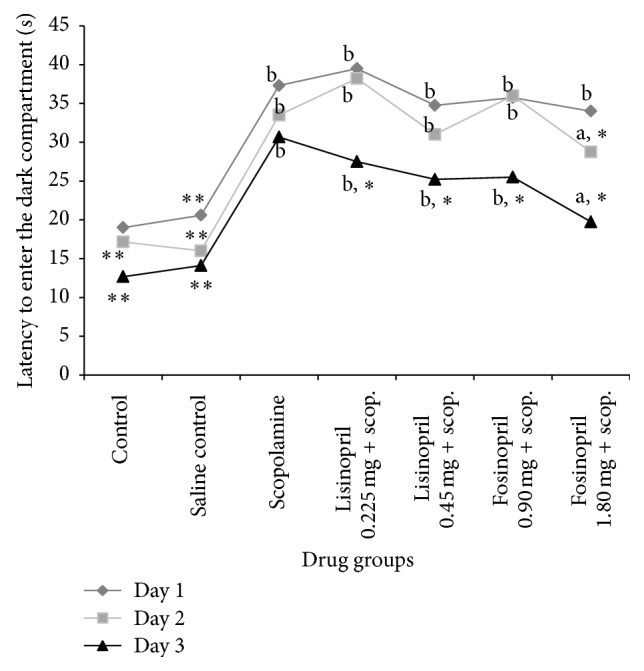
Effect of lisinopril and fosinopril in scopolamine-induced amnesic rats during the exploration trials in passive avoidance test. Comparison between control, scopolamine, lisinopril, and fosinopril during the exploration trials in passive avoidance test. Values are mean ± SE, ^*∗*^versus scopolamine (*p* < 0.05), ^*∗∗*^versus scopolamine (*p* < 0.001), ^a^versus control (*p* < 0.05), and ^b^versus control (*p* < 0.001).

During the exploratory trials, the latency to enter the dark compartment was decreased in all the groups from first to third trial. The scopolamine treated animals took more time to enter the dark compartment during the three exploration trials (Table 2 and Figure 1). Rats pretreated with lower and higher doses of lisinopril did not show significant difference during the exploration trials compared to scopolamine treated rats. Lower dose of fosinopril also could not significantly ameliorate scopolamine-induced learning impairment as reflected in their latency during the exploratory trials (Table 2 and Figure 1). However, rats pretreated with high dose of fosinopril showed a decreased latency to enter the dark compartment during the exploratory trials and spent more time in the light compartment in each successive trial compared to scopolamine treated rats (*p* < 0.05). This is indicative of positive learning behaviour among fosinopril treated rats.

During the memory retention test, the latency to enter the dark compartment was significantly reduced for scopolamine and lisinopril treated groups compared to normal control rats (*p* < 0.001). Rats which received scopolamine and lisinopril also spent lesser time in the light compartment compared to normal control rats (*p* < 0.05). Pretreatment with higher dose of fosinopril increased the entrance latency time of rats and the difference was statistically significant compared to scopolamine group (*p* < 0.001). Rats pretreated with higher dose of fosinopril also spent more time in the light compartment indicating improved memory retention and the difference was significant compared to rats that received only scopolamine (*p* < 0.001), as shown in Table 3.

### 3.3. Effects of Lisinopril and Fosinopril during Morris Water Maze Test

**Figure 2:**
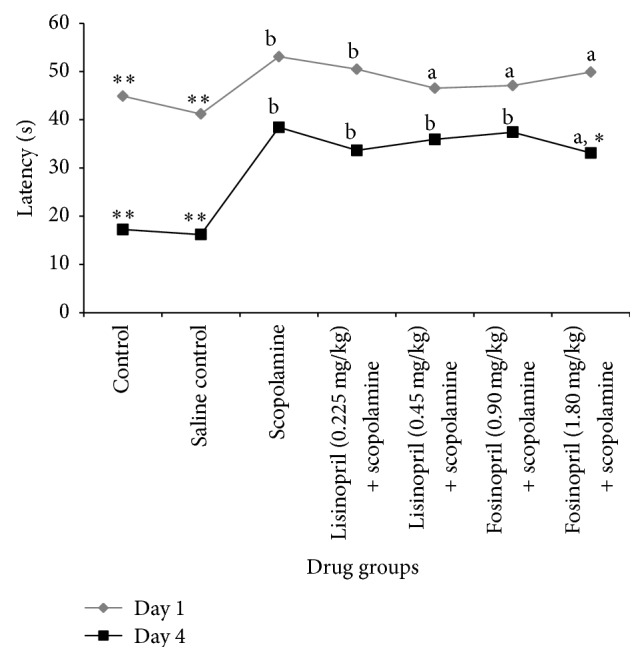
Effect of lisinopril and fosinopril in scopolamine-induced amnesic rats during the acquisition trials in Morris water maze test. Comparison between control, scopolamine, lisinopril, and fosinopril treated groups during the acquisition trials on day 1 and day 4 of Morris water maze test. Values are mean ± SE, ^*∗*^versus scopolamine (*p* < 0.05), ^*∗∗*^versus scopolamine (*p* < 0.001), ^a^versus control (*p* < 0.05), and ^b^versus control (*p* < 0.001).

Control rats which received gum acacia and saline rapidly learned the location of the hidden platform as reflected by a decrease in their latencies from day 1 to day 4, indicating normal acquisition behaviour (Table 4 and Figure 2). Rats which received scopolamine showed an increased latency to locate the hidden platform during the acquisition trials, the difference being statistically significant compared to control rats (*p* < 0.001). This indicates impairment of acquisition in the scopolamine treated rats. Further, scopolamine treated rats showed a significant increase in the latency to locate the target quadrant (Q4) compared to control rats (*p* < 0.001), which indicates impaired memory (Table 4 and Figure 2).

Rats treated with lisinopril (0.225 mg/kg and 0.45 mg/kg) and low dose fosinopril (0.90 mg/kg) could not ameliorate the scopolamine-induced impairment in both the learning and memory retention parameters of water maze tests (Tables 4 and 5). This was evident by increased latency to locate the hidden platform during the acquisition trials and greater time spent to locate the target quadrant (Q4) during the memory retention trial. Higher dose of fosinopril (1.80 mg/kg) showed a decrease in the latencies from day 1 to day 4 during the acquisition trials. Pretreatment with fosinopril in higher dose demonstrated a reversal of amnesia, as indicated by decreased latency to reach the target quadrant and increased time spent in target quadrant, the difference being significant compared to scopolamine (*p* < 0.05).

### 3.4. Effects of Lisinopril and Fosinopril on Hippocampal Morphology and Degree of Neuronal Survival

**Figure 3:**
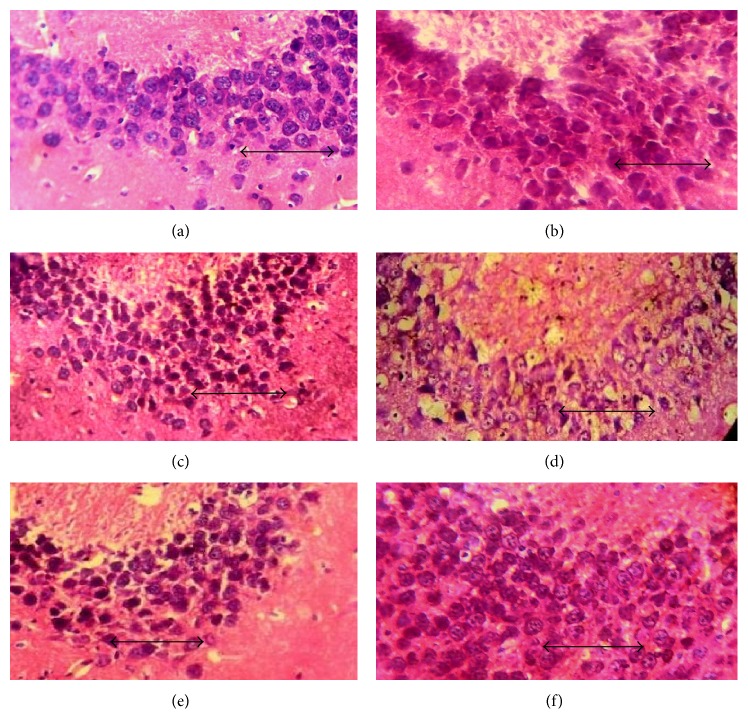
Effect of lisinopril and fosinopril on the morphology of CA3 region of hippocampus in scopolamine-induced amnesic rats. Light photomicrographs of CA3 layer of hippocampus in (a) control rats, (b) scopolamine, (c) lisinopril (0.225 mg/kg) + scop., (d) lisinopril (0.45 mg/kg) + scop., (e) fosinopril (0.90 mg/kg) + scop., and (f) fosinopril (1.80 mg/kg) + scop. Scale bar represents 1 *µ*.

**Figure 4:**
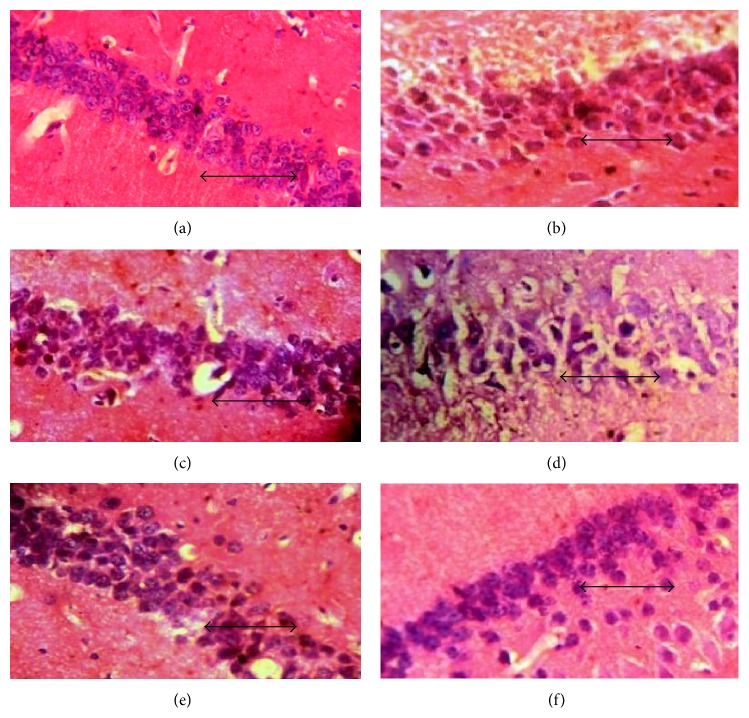
Effect of lisinopril and fosinopril on the morphology of CA1 region of hippocampus in scopolamine-induced amnesic rats. Light photomicrographs of CA1 layer of hippocampus in (a) control rats, (b) scopolamine, (c) lisinopril (0.225 mg/kg) + scop., (d) lisinopril (0.45 mg/kg) + scop., (e) fosinopril (0.90 mg/kg) + scop., and (f) fosinopril (1.80 mg/kg) + scop. Scale bar represents 1 *µ*.

**Figure 5:**
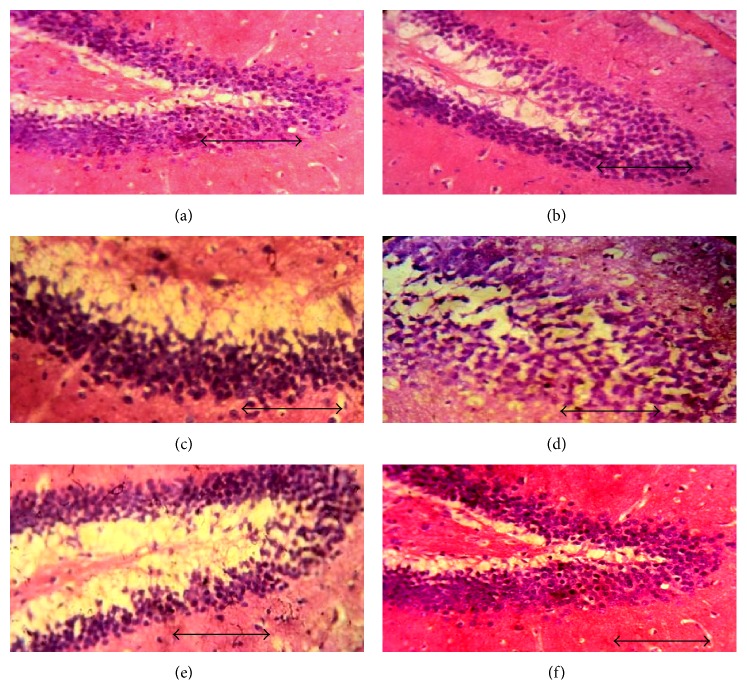
Effect of lisinopril and fosinopril on the morphology of dentate gyrus region of hippocampus in scopolamine-induced amnesic rats. Light photomicrographs of dentate gyrus layer of hippocampus in (a) control rats, (b) scopolamine, (c) lisinopril (0.225 mg/kg) + scop., (d) lisinopril (0.45 mg/kg) + scop., (e) fosinopril (0.90 mg/kg) + scop., and (f) fosinopril (1.80 mg/kg) + scop. Scale bar represents 1 *µ*.

In hematoxylin and eosin stained sections of hippocampal CA3 (Figure 3), CA1 (Figure 4), and dentate gyrus regions (Figure 5), cells with lightly stained nucleus, healthy cell membrane, and clear cytoplasm were considered as normal neurons while flame-shaped cells with pyknotic cell bodies, homogenous cytoplasm, and intense basophilic appearance were considered as damaged cells. Control rats and rats treated with higher dose of fosinopril demonstrated healthy neurons in all the three regions of the hippocampus compared to scopolamine treated rats which showed damaged neuronal cells. However, treatment with lower and higher doses of lisinopril could not markedly reverse the scopolamine-induced morphological changes produced in the hippocampus as demonstrated in Figures 3, 4, and 5.

**Figure 6:**
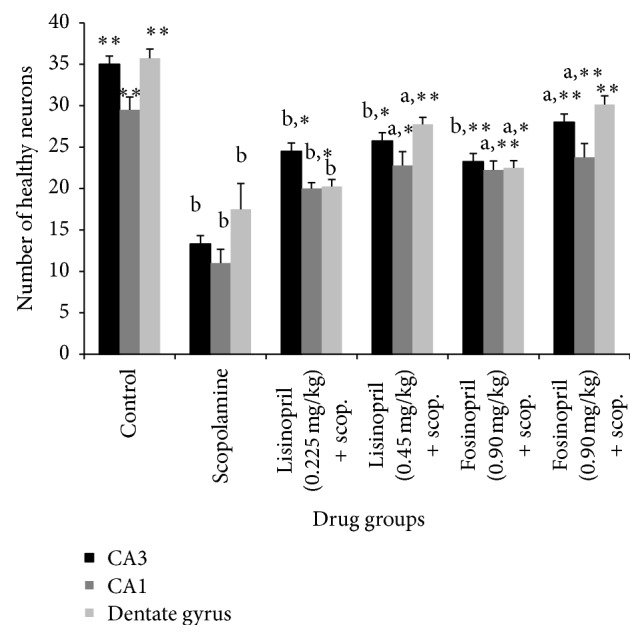
Number of healthy neurons at CA3, CA1, and dentate gyrus regions of the hippocampus of scopolamine treated amnesic rats following treatment with lisinopril and fosinopril. Comparison between control, scopolamine, lisinopril, and fosinopril treated groups in the probe trial of Morris water maze test in which no platform was present. Values are mean ± SE, ^*∗*^versus scopolamine (*p* < 0.05), ^*∗∗*^versus scopolamine (*p* < 0.001), ^a^versus control (*p* < 0.05), and ^b^versus control (*p* < 0.001).

Quantification of healthy neurons in the CA3, CA1, and dentate gyrus (DG) regions revealed a significant decrease in the mean number of neurons in the scopolamine group compared to control rats (Figure 6). The mean number of healthy surviving neurons in the CA3, CA1, and DG of control group was found to be 35.00 ± 1.29, 29.50 ± 1.55, and 35.75 ± 1.10, respectively. This was reduced to 13.30 ± 1.37, 11.0 ± 1.68, and 17.5 ± 3.12 for CA3, CA1, and DG regions, respectively. ANOVA test revealed a significant difference in the mean values of control group compared to scopolamine group (^*∗∗*^ *p* < 0.001) as shown in Figure 6. In rats pretreated with higher dose of lisinopril (0.45 mg/kg), the mean number of surviving neurons was 25.75 ± 1.79, 22.78 ± 1.69, and 27.75 ± 0.85 in the CA3, CA1, and DG regions, respectively, while for rats that received higher dose of fosinopril (1.80 mg/kg), the mean number of surviving neurons was 28.0 ± 1.49, 23.76 ± 1.68, and 30.15 ± 1.04 in CA3, CA1, and DG regions, respectively. Higher dose of lisinopril and fosinopril showed a significantly increased number of healthy neurons compared to scopolamine group but the numbers were not significantly increased compared to control rats (^b^ *p* < 0.001 and ^a^ *p* < 0.05 for lisinopril and fosinopril, resp.) as shown in Figure 6.

## 4. Discussion

The present study was carried out using rats for investigation of learning and memory tasks. Passive avoidance is a fear-aggravated task used to assess memory or retention in animal models of CNS disorders, particularly dementia. Rats, as a part of their normal behaviour, generally avoid bright illumination and prefer dim illumination. When placed in a brightly illuminated compartment connected with a dark enclosure, they rapidly enter the dark compartment and remain there [15]. Once they receive an aversive consequence (foot shock) in the dark compartment, the animals modify their behaviour to avoid a noxious event by suppressing the learned habits of staying in the dark compartment and remain in the bright compartment. Since there is punishment to the natural exploratory drive of a rodent with a pulsating electric foot shock, this is clearly an aversive task. In our present study, administration of scopolamine clearly produced memory deficits (amnesia) in rat performance in passive avoidance test as indicated by their shorter latency to enter into the dark compartment in the memory retention test compared to the control group. The mean latency of rats treated with high dose of fosinopril (1.80 mg/kg) was significantly higher compared to scopolamine group, indicating reversal of amnesia. This showed that scopolamine treated rats after being exposed to aversive stimulation in the passive avoidance task failed to remember the task on the following day, but this effect could be attenuated following treatment with fosinopril at a dose of 1.80 mg/kg, indicating that fosinopril has a positive effect on memory retention.

The Morris water maze (MWM) test is a well-established model for evaluating hippocampal dependent memory deficits in experimental animals and has been used for the evaluation of drugs with neurocognitive enhancing ability [18]. In the MWM task, the animal learns to swim in a water tank, guided by external cues, and climbs up to a submerged platform [16]. Based upon spatial information, this animal learns how to escape to a platform. Rats and mice are natural swimmers, but in this task they just want to get out of the water and escape into the platform. In our study, administration of scopolamine produced severe deficits in both the acquisition and the memory retention trials as indicated by their longer latencies to escape into the submerged platform. Scopolamine treated animals also spent lesser time in the target quadrant during the retention trial compared to control rats. Treatment with fosinopril 1.80 mg/kg could attenuate the scopolamine-induced memory deficits in the water maze test, demonstrated by their shorter latencies to locate the hidden platform during the acquisition and longer time spent in the target quadrant during the retention, thus indicating its potential memory enhancing effects.

The elevated plus maze test has been considered as an indicator of short-term memory [14]. In this test, scopolamine treated rats showed a significant decrease in transfer latencies on 1st day and 2nd day which could be markedly attenuated by pretreatment with fosinopril 1.80 mg/kg.

Data generated from the present study demonstrated that administration of scopolamine induces profound memory deficits in rat performance in all the three paradigms of learning and memory tasks. This change in behaviour was found to be associated with signs of neurodegeneration in the hippocampus as evident by the deeply stained and shrunken neuronal cells in CA3, CA1, and dentate gyrus regions of the hippocampus. Administration of 1.80 mg/kg fosinopril was found to arrest the scopolamine-induced degenerative changes in the hippocampus, as reflected by the decreased number of damaged neuronal cells in all the three regions of the hippocampus. Although the degeneration of cells in the hippocampus induced by scopolamine was decreased in all the groups, marked differences were noted only in rats that were treated with higher dose (1.80 mg/kg) of fosinopril.

Our study is the first of its kind that has investigated the effect of fosinopril on behavioural paradigms of memory retention. The beneficial effect of fosinopril on memory retention could be partly attributed to its ability to suppress angiotensin-II (Ang-II) mediated inhibition of acetylcholine (ACh) release in the brain [19]. The brain is known to possess an intrinsic renin angiotensin system (RAS) that is involved in memory and cognition [20] and the brain Ang-II is involved in inhibiting release of ACh. ACh is the primary neurotransmitter involved in learning and memory [19] and reductions in brain ACh level have been found to strongly correlate with the degree of cognitive impairment in patients with AD [21]. The integrity of cholinergic system is essential to learning and memory, and scopolamine, a muscarinic receptor antagonist, can produce learning and memory defects by disrupting the functional integrity of the cholinergic system through competitive receptor blockade [2]. Thus, a drug such as fosinopril that can reverse scopolamine-induced behavioural deficits by enhancing cholinergic transmission is likely to offer beneficial effects which may improve debilitated patient's condition in AD.

The memory deficits produced by scopolamine in the behavioral tasks could also be due to the altered functioning of neurons in both the hippocampal and the amygdala. It is well-established that structural abnormalities of hippocampus, cortex, and medial temporal lobe structures along with a decrease in hippocampal volume are associated with the severity of deficits in learning and memory [22, 23]. In our study, the hematoxylin and eosin staining of the hippocampal region in scopolamine group clearly showed damaged neuronal cells indicating the degenerative changes in these areas. Further, scopolamine treated rats showed lesser number of healthy neurons compared to control and drug treated groups, indicating loss of neuronal function. The exact mechanism responsible for this degeneration is not clear but it could be due to the generation of reactive oxygen species. In previous reports, scopolamine has been shown to trigger the induction of ROS and cause free radical injuries associated with reduced activity of antioxidant enzymes like superoxide dismutase glutathione peroxidase in the brain [24]. In the current study, fosinopril was able to attenuate the hippocampal damage caused by scopolamine as reflected by an increase in the number of healthy neurons and decrease in the number of damaged neuronal cells in the CA3 and dentate gyrus regions. Although the precise mechanism by which fosinopril has produced these beneficial effects is not known, it could be attributed to its effect on ACh release or to its antioxidant property. A study by Hayek et al. [25] reported that ACEIs exhibit antioxidant properties and block LDL oxidation, lipid peroxidation, and the generation of MDA and 4-HNE. Furthermore, ACE inhibitors such as captopril and fosinopril have been found to exhibit a potent antiatherogenic effect in apoE−/− mice due to their protective effect against LDL oxidation [25, 26]. An improvement in cerebral blood flow could also be a factor involved in mediating the memory enhancing effects by fosinopril though other putative mechanisms cannot be ruled out.

The present study thus showed that, among the two centrally acting ACEIs, fosinopril but not lisinopril exhibits antiamnesic activity. Lack of significant antiamnesic activity with lisinopril could be due to its poor lipophilicity, resulting in their lesser concentration in the brain [27]. Both fosinopril and lisinopril are centrally acting ACEIs with an ability to cross the blood brain barrier [28, 29]. However, differences in lipophilicity between lisinopril and fosinopril could be responsible for the differences in their degree of penetration into the brain. Lipophilicity is an important physicochemical property that governs the passage of drugs across cells and tissues. Higher the lipophilicity of the drug, better the tissue and cell penetration. Differences in lipophilicity between lisinopril and fosinopril could be attributed to their heterogeneous chemical structure [30]. Lisinopril, like enalapril, contains pyrrolidone ring of proline, whereas fosinopril contains a bicyclic ring that accounts for its higher lipophilicity. Fosinopril which is more lipophilic exhibits a plasma protein binding of more than 90% whereas lisinopril which is least lipophilic exhibits minimal protein binding. The more lipophilic compound exhibits a greater degree of plasma protein binding, which in turn increases its ACE inhibitory activity in various tissues [30]. Thus, better efficacy seen with fosinopril could be due to its ability to penetrate the brain to a greater degree than lisinopril though other factors need to be evaluated. Differences in pharmacological and physicochemical properties such as lipophilicity, tissue penetration, absolute bioavailability, and plasma half-life extend to differences in the efficacy among various ACEIs [31]. Thus, members of a drug class although having a common mechanism of action, they may not be identical in their pharmacodynamic efficacies due to marked differences in their chemical structure and pharmacokinetic features.

In a previous study, lisinopril has been shown to reverse the memory deficits in streptozotocin-induced experimental dementia [32]. In contrast,* in vitro* findings from an earlier report suggested that the ACEI lisinopril could interfere with the ability of ACE to inhibit the aggregation of A*β* and reduce A*β* mediated toxic effects in rat cells, further worsening the memory deficits in rats [33]. The differences in responses remain unexplained, but it could be due to differences in experimental designs between the studies such as the age of the animal and the period for which the treatment was received or due to differences in the species involved.

In conclusion, our study demonstrated that centrally acting ACEI such as fosinopril has potent memory, enhancing effects against scopolamine-induced amnesic mice. Since ACEIs are one of the commonly used drugs for hypertension, treatment with fosinopril may be particularly beneficial in preventing the memory deficits in elderly patients with both hypertension and dementia. However, further studies will be required to investigate the other putative mechanisms by which fosinopril may exert its beneficial effect on cognition.

## Figures and Tables

**Table 1:** Effects of lisinopril and fosinopril on the transfer latencies on 1st day and 2nd day in elevated plus maze test.

Treatment	Transfer latency on the 1st day (sec)	Transfer latency on 2nd day after 24 h (sec)
Control	57.50 ± 4.32^*∗∗*^	35.50 ± 2.15^*∗∗*,b^
Saline control	61.00 ± 3.20^*∗∗*^	37.90 ± 2.81^*∗∗*^
Scopolamine	92.17 ± 5.57^b^	94.00 ± 3.55^b^
Lisinopril (0.225 mg/kg) + scopolamine	83.33 ± 3.81^b^	75.61 ± 2.75^b^
Lisinopril (0.45 mg/kg) + scopolamine	76.16 ± 5.74^*∗*,a^	55.17 ± 3.04^*∗∗*,a^
Fosinopril (0.90 mg/kg) + scopolamine	85.33 ± 3.56^b^	75.67 ± 5.16^b^
Fosinopril (1.80 mg/kg) + scopolamine	73.50 ± 3.34^*∗∗*,a^	53.00 ± 3.67^*∗∗*^

Comparisons between control, scopolamine, lisinopril, and fosinopril treated groups in the elevated plus maze test. Values are mean ± SE, ^*∗*^versus scopolamine (*p* < 0.05), ^*∗∗*^versus scopolamine (*p* < 0.001), ^a^versus control (*p* < 0.05), and ^b^versus control (*p* < 0.001).

**Table 2:** Effects of lisinopril and fosinopril on the exploratory behaviour of scopolamine-induced amnesic rats in passive avoidance test.

Treatment	Exploration trial (day 1)	Exploration trial (day 2)	Exploration trial (day 3)
Control	19.3 ± 0.95^*∗∗*^	17.16 ± 1.54^*∗∗*^	12.67 ± 1.31^*∗∗*^
Saline control	20.6 ± 1.28^*∗∗*^	16.00 ± 2.71^*∗∗*^	14.10 ± 1.92^*∗∗*^
Scopolamine	37.3 ± 2.55^b^	33.5 ± 1.88^b^	30.67 ± 1.85^b^
Lisinopril (0.225 mg/kg) + scopolamine	39.5 ± 2.91^b^	38.22 ± 1.03^b^	27.50 ± 2.46^b^
Lisinopril (0.45 mg/kg) + scopolamine	34.75 ± 1.88^b^	31.00 ± 2.71^b^	25.21 ± 1.18^b,*∗*^
Fosinopril (0.90 mg/kg) + scopolamine	35.75 ± 2.78^b^	36.0 ± 2.92^b^	25.50 ± 1.08^b,*∗*^
Fosinopril (1.80 mg/kg) + scopolamine	34.00 ± 3.02^b^	28.75 ± 2.45^a,*∗*^	19.75 ± 1.26^a,*∗*^

Comparison between control, scopolamine, lisinopril, and fosinopril during the exploration trials in passive avoidance test. Values are mean ± SE, ^*∗*^versus scopolamine (*p* < 0.05), ^*∗∗*^versus scopolamine (*p* < 0.001), ^a^versus control (*p* < 0.05), and ^b^versus control (*p* < 0.001).

**Table 3:** Effects of lisinopril and fosinopril on memory retention behaviour of scopolamine-induced amnesic rats in passive avoidance test.

Treatment	Latency to enter the dark compartment (sec) 24 h after receiving foot shock	Total time spent in light compartment (sec) 24 h after receiving foot shock
Control	51.83 ± 2.71^*∗∗*^	114.50 ± 3.87^*∗∗*^
Saline control	49.29 ± 2.21^*∗∗*^	109.78 ± 2.55^*∗∗*^
Scopolamine	14.83 ± 1.05^b^	57.33 ± 4.23^b^
Lisinopril (0.225 mg/kg) + scopolamine	25.68 ± 2.34^b^	63.00 ± 5.45^b^
Lisinopril (0.45 mg/kg) + scopolamine	27.81 ± 4.78^b^	73.67 ± 2.24^a^
Fosinopril (0.90 mg/kg) + scopolamine	29.83 ± 4.09^*∗*,b^	88.66 ± 8.59^b^
Fosinopril (1.80 mg/kg) + scopolamine	36.33 ± 2.89^*∗∗*,a^	98.17 ± 3.54^*∗∗*^

Comparisons between control, scopolamine, lisinopril, and fosinopril during the retention trial in passive avoidance test. Values are mean ± SE, ^*∗*^versus scopolamine (*p* < 0.05), ^*∗∗*^versus scopolamine (*p* < 0.001), ^a^versus control (*p* < 0.05), and ^b^versus control (*p* < 0.001).

**Table 4:** Effects of lisinopril and fosinopril during acquisition trials in Morris water maze test.

Treatment	Latency (sec) to locate the hidden platform on day 1	Latency (sec) to locate the hidden platform on day 4	Mean swim speed (seconds)
Control	44.89 ± 1.42^*∗∗*^	17.24 ± 0.88^*∗∗*^	0.166 ± 0.12^*∗∗*^
Saline control	41.20 ± 1.88^*∗∗*^	16.18 ± 1.71^*∗∗*^	0.172 ± 0.22^*∗∗*^
Scopolamine	53.08 ± 1.56^b^	38.42 ± 2.59^b^	0.387 ± 0.18^b^
Lisinopril (0.225 mg/kg) + scopolamine	50.47 ± 1.45^b^	33.64 ± 1.90^b^	0.307 ± 0.07^b^
Lisinopril (0.45 mg/kg) + scopolamine	46.53 ± 2.08^a^	35.93 ± 1.43^b^	0.268 ± 0.20^b^
Fosinopril (0.90 mg/kg) + scopolamine	47.08 ± 2.58^a^	37.42 ± 1.23^b^	0.294 ± 0.17^b^
Fosinopril (1.80 mg/kg) + scopolamine	49.87 ± 0.89^a^	33.10 ± 1.45^*∗*,a^	0.198 ± 0.18^a,*∗*^

Comparisons between control, scopolamine, lisinopril, and fosinopril treated groups during acquisition trials on day 1 and day 4, and on the mean swim speed during the Morris water maze test. Values are mean ± SE, ^*∗*^versus scopolamine (*p* < 0.05), ^*∗∗*^versus scopolamine (*p* < 0.001), ^a^versus control (*p* < 0.05), and ^b^versus control (*p* < 0.001).

**Table 5:** Effects of lisinopril and fosinopril in the probe trial on day 5 of Morris water maze test.

Treatment	Latency to enter the target quadrant (sec)	Total time spent in target quadrant (sec)
Control	14.59 ± 1.54^*∗∗*^	26.11 ± 0.54^*∗∗*^
Saline control	17.11 ± 1.62^*∗∗*^	29.20 ± 1.66^*∗∗*^
Scopolamine	27.48 ± 2.00^b^	12.58 ± 0.42^b^
Lisinopril (0.225 mg/kg) + scopolamine	23.90 ± 1.37^b^	12.28 ± 0.37^b^
Lisinopril (0.45 mg/kg) + scopolamine	22.83 ± 1.96^b^	14.75 ± 0.96^b^
Fosinopril (0.90 mg/kg) + scopolamine	22.29 ± 1.91^b^	14.37 ± 0.91^b^
Fosinopril (1.80 mg/kg) + scopolamine	20.18 ± 0.85^*∗*,a^	19.25 ± 1.85^*∗*,a^

Comparison between control, scopolamine, lisinopril, and fosinopril treated groups in the probe trial of Morris water maze test in which no platform was present. Values are mean ± SE, ^*∗*^versus scopolamine (*p* < 0.05), ^*∗∗*^versus scopolamine (*p* < 0.001), ^a^versus control (*p* < 0.05), and ^b^versus control (*p* < 0.001).
